# Development of Vogt–Koyanagi–Harada disease-like uveitis during treatment by anti-programmed death-1 antibody: a case report

**DOI:** 10.1186/s12886-024-03484-9

**Published:** 2024-06-07

**Authors:** Jia-ning Wang, Yue Zhang, Chen-ye Huang, Kang Li, Xiao-bing Yu

**Affiliations:** 1 Department of Ophthalmology, Beijing Hospital, National Center of Gerontology, Institute of Geriatric Medicine, Chinese Academy of Medical Sciences, Beijing, China; 2 Peking University Fifth School of Clinical Medicine, Beijing, China

**Keywords:** Immune checkpoint inhibitors (ICIs), Vogt-Koyanagi-Harada disease (VKHD)-like uveitis, Immune-related adverse events (irAEs), Anti-PD-1 (Toripalimab)

## Abstract

**Background:**

Several immune checkpoint inhibitors (ICIs) have been linked to the occurrence of Vogt-Koyanagi-Harada disease (VKHD)-like uveitis. Among the ICIs, there has been no report of immune-related adverse events (irAEs) caused by a new programmed death protein-1(PD-1) monoclonal antibody (Toripalimab).

**Case presentation:**

This paper presents a case of VKHD-like uveitis that arose following Toripalimab therapy for urothelial cancer of the bladder, and the patient experienced symptoms 10 days after the final dosage of 20 months of medication treatment. This patient with bladder uroepithelial carcinoma had severe binocular acute panuveitis with exudative retinal detachment after receiving Toripalimab therapy. Binocular VKHD-like uveitis was suggested as a diagnosis. Both eyes recovered after discontinuing immune checkpoint inhibitors and local and systemic corticosteroid treatment.

**Conclusions:**

This report suggests that VKHD-like uveitis can also occur in patients receiving novel PD-1 antibodies and the importance of paying attention to eye complications in patients receiving treatment over a long period.

## Background

The first domestic anti-PD-1 monoclonal antibody with fully independent intellectual property rights in China is Toripalimab (TuoyiTM), which has been approved for use in treating melanoma, nasopharyngeal cancer, urothelial carcinoma, and other conditions [1]. Immune checkpoint inhibitors (ICIs) treat systemic cancers primarily by blocking one of three ligands, including cytotoxic T lymphocyte antigen-4 (CTLA-4), programmed death protein-1 (PD-1), and programmed death ligand-1 [2], that trigger T lymphocytes to activate and assault malignant cells. Immune checkpoint inhibitors are becoming an effective tool in the fight against cancer. Both early and late stages of cancer can have long-lasting responses to treatment [3].

Review of the literature, that no cases of VKHD-like uveitis have been reported following therapy with Toripalimab, an innovative anti-PD-1 antibody. This is the first report of a new PD-1 monoclonal antibody. The goal of this study is to disclose our findings in a case with VKHD-like uveitis that emerged following the start of anti-PD-1 antibody treatment for bladder urothelial cancer.

## Case presentation

A 69-year-old Asian elderly male was admitted to the emergency department of another hospital on June 24, 2022, after suffering from eye pain accompanied by vision loss for one day. His best correct visual acuity (BCVA) was 20/200 in both eyes, and his intraocular pressure was 49 mmHg in the right eye and 48 mmHg in the left. After emergency therapy for intraocular pressure lowering, the patient’s symptoms decreased. The patient had no prior ocular history, and his BCVA was 20/20.

**Fig. 1:**
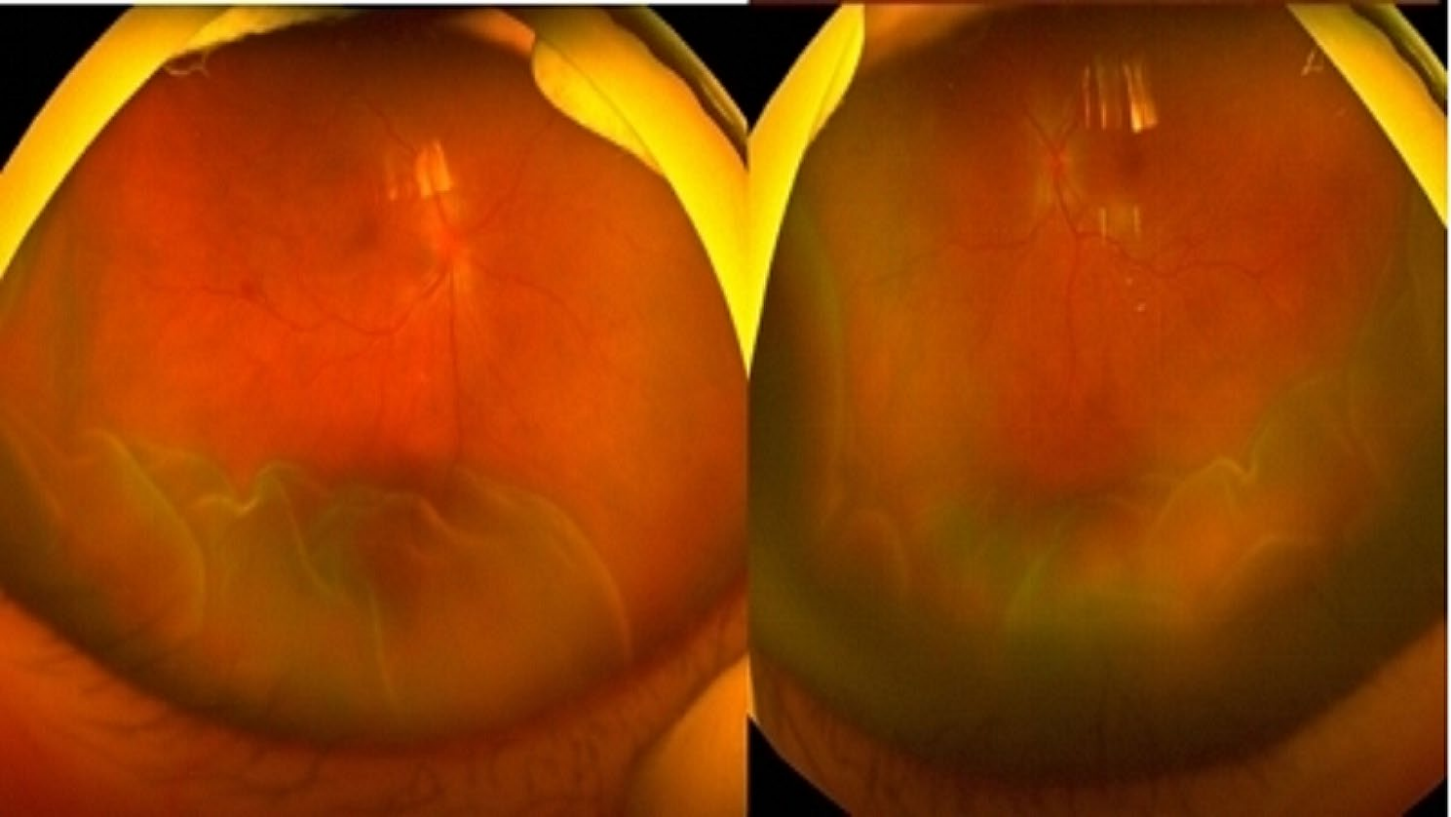
Fundus color photos on admission: edema of the optic disc in both eyes, peripheral retina exudative bulge detachment

**Fig. 2:**
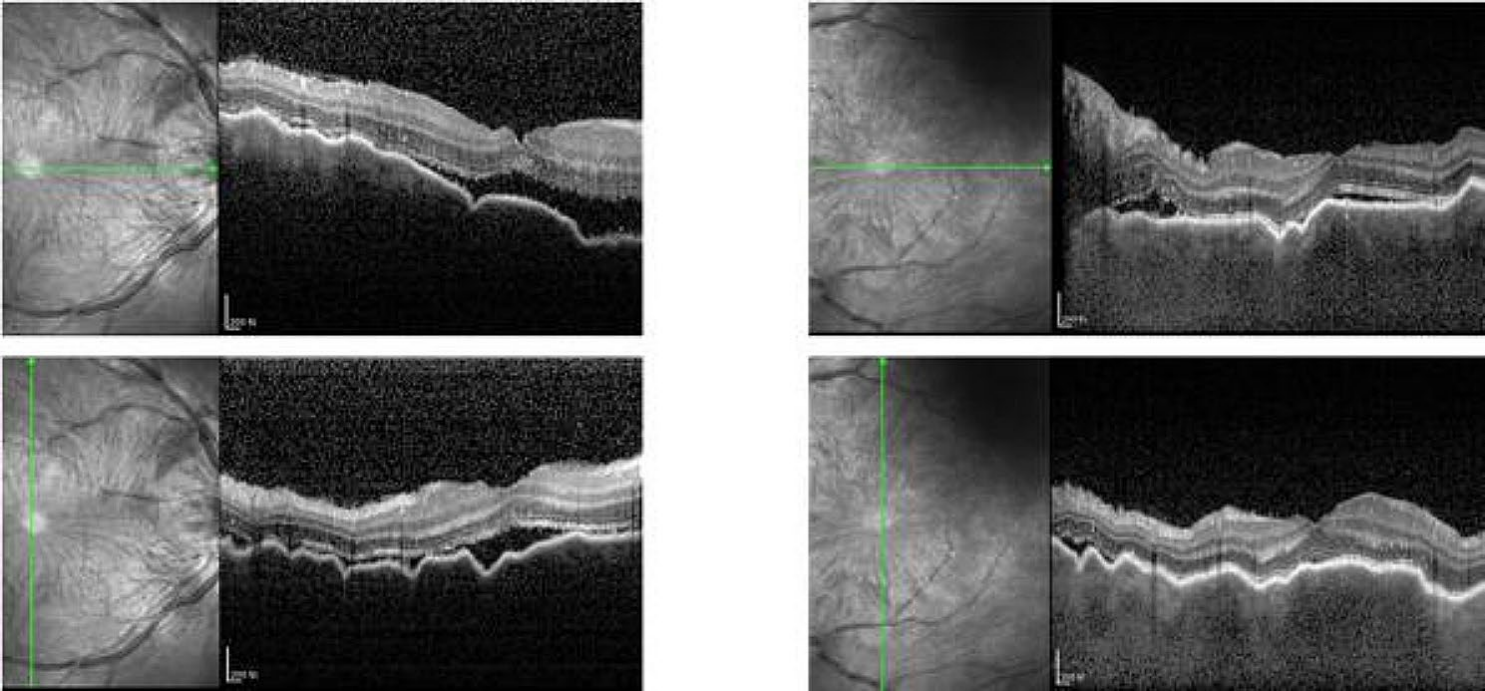
Optical Coherence Tomography (OCT): choroidal folds and plasma retinal detachment in both eyes

**Fig. 3:**
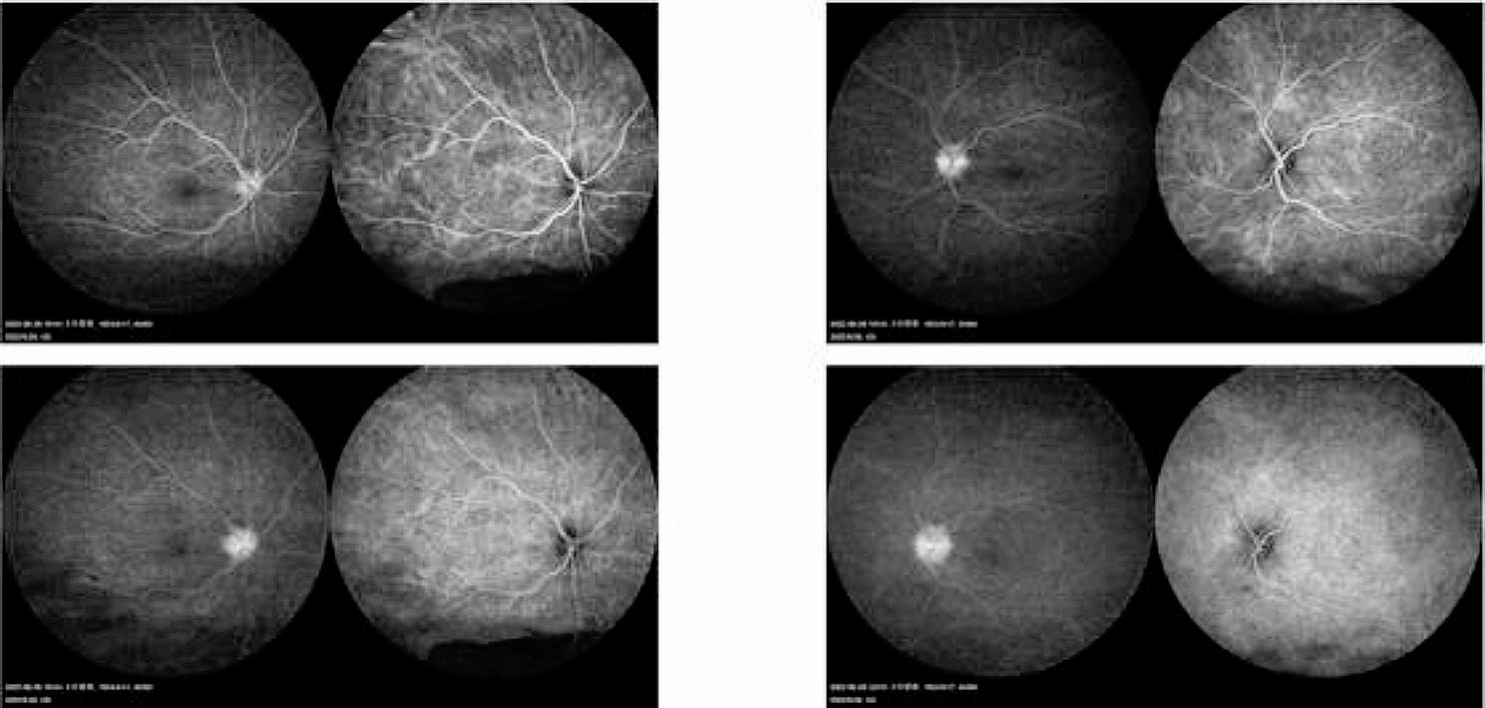
Fluorescein Fundus Angiography (FFA)& Indocyanine Green Angiography (ICGA): punctate hyper fluorescence of optic discs in both eyes, no retinal leakage

The next day, the patient presented to our outpatient clinic, but this time his BCVA was counting fingers in both eyes, and intraocular pressure was 6 mmHg in both eyes. Physical examination revealed mild conjunctival congestion in both eyes, mild corneal edema, shallow anterior chambers, moderate inflammatory cells in the anterior chamber, dilated and fixed pupil, posterior synechiae, lens opacity, and pigmentation on the anterior capsule of the lens. Binocular fundus examination revealed optic disc swelling in both eyes, peripheral retinal serous detachments, and no retinal tears (Fig. 1). On optical coherence tomography (OCT) the choroid was swollen and there was a bacillary layer serous retinal detachment in both eyes (Fig. 2). In both eyes, late fundus fluorescein angiography showed pin-point hyperfluorescence (Fig. 3). The results of laboratory testing for infection and autoimmunity were negative for antinuclear antibody (ANA), anti-double-stranded DNA antibody (ds-DNA), erythrocyte sedimentation rate (ESR), C-reactive protein (CRP), hepatitis B surface antigen (HBsAg), syphilis antibody, HIV antibody, hepatitis C antibody, tuberculosis infection cell spot test (T-SPOT), and other tests. Based on the patient’s test findings and the absence of any systemic signs and symptoms, infectious uveitis with VKH was ruled out.

The patient underwent surgical treatment for bladder urothelial carcinoma in May 2020. Due to poor response to chemotherapy(Gemcitabine and Cisplatin), he was treated with a combination of Toripalimab three months later and got a total of 19 rounds of Toripalimab therapy for 18 months until March 2022. Because of a cancer recurrence, the patient underwent a second surgery in March 2022 and was treated with Toripalimab once after surgery. It had been 10 days since Toripalimab therapy when she was admitted.

According to the patient’s history of treatment with an immune checkpoint inhibitor, combined with the patient’s clinical symptoms and signs, the patient was eventually diagnosed with VKHD-like uveitis after continuing to consult relevant literature.

**Fig. 4:**
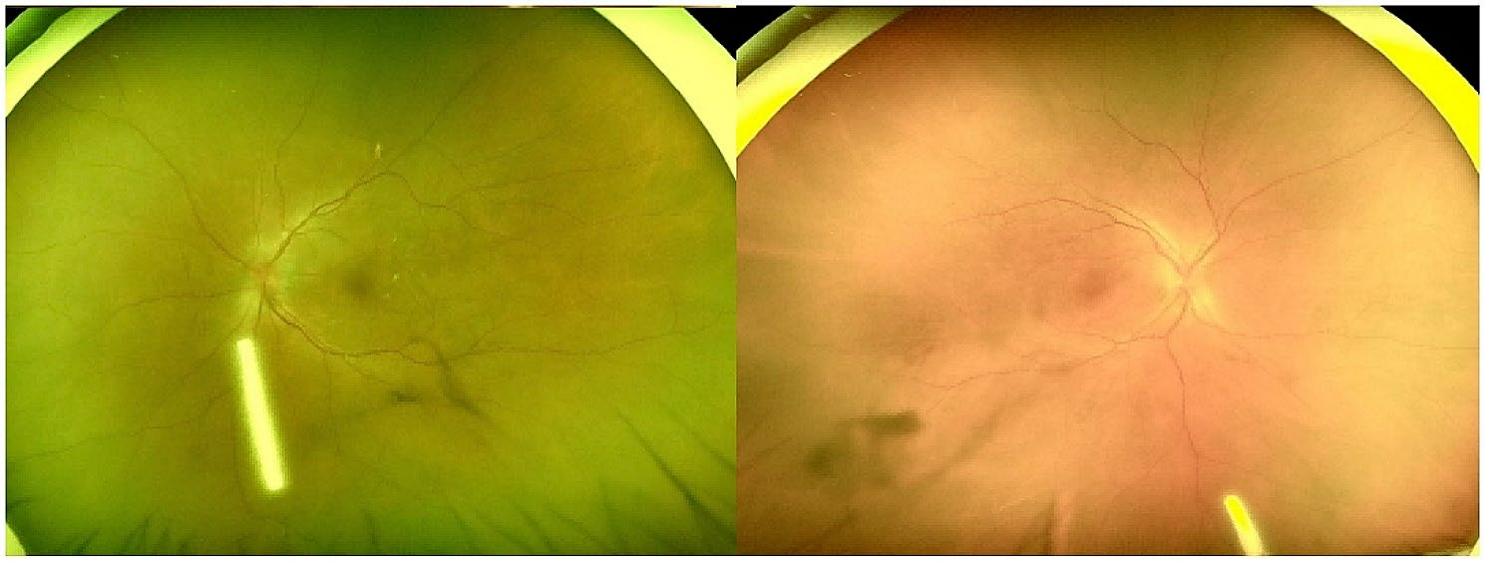
Fundus color photograph after 5 days of treatment: edema of optic discs in both eyes was reduced compared to the previous one, retinal flatness and flatness of retinas in both eyes, and rod-shaped dexamethasone retardant was seen in the vitreous cavity of both eyes

According to the guidelines and consultations with experts from the departments of oncology, immunology, and ophthalmology, it was decided to discontinue the use of Toripalimab and instead give patients a dexamethasone sustained release implant of both eyes, as well as an oral prednisone tablet of 1 mg per kilogram of body weight (60 mg) as the starting dose for 2 weeks, and gradually reduce the dose depending on the situation. After 5 days of treatment, the patient’s vision improved significantly. The right eye BCVA was 20/80, and the left eye BCVA was 20/50. The patient was released from the hospital with binocular exudative retinal detachment (Fig. 4). The patient was reexamined after taking a 60-mg prednisone pill orally for two weeks, and the fundus of both eyes was stable. The patient was instructed to limit his oral steroids to 10 mg per week. After three weeks, the patient’s BCVA had improved to 20/25, and the retina in both eyes was flat. At this time, the patient’s oral corticosteroid dose is 30 mg, and the amount can be gradually reduced, with a 5 mg reduction every 10 days. There were no symptoms of active uveitis in both eyes at the time of the last review (January 2023), and the retinas were flat in both eyes.

## Discussion and conclusion

Toripalimab was used to treat VKHD-like uveitis after surgery for urothelial carcinoma of the bladder, with a period of more than 20 months from therapy to symptom manifestation. This patient had one of the longest gaps between the start of ICIs medication and the development of VKHD-like uveitis among documented instances.

Immune checkpoint inhibitors have emerged as a potent strategy in the treatment of malignancies. Despite the remarkable efficiency of immune checkpoint inhibitors in tumor therapy, there are unavoidable side effects that induce immunological dysfunction, known as immune-related adverse events (irAEs) [4]. irAEs can cause fatigue, pruritus, diarrhea, and eye side effects, sometimes leading to severe conditions such as immune-related pneumonia, immune-related myocarditis, immune-related neurotoxicity, and colitis. Among the common eye side effects are dry eye, uveitis, vision loss, and myasthenia gravis with ocular involvement. About 20 individuals have been diagnosed with Vogt-Koyanagi-Harada disease (VKHD)-like uveitis, and these patients are being treated with ICIs for malignant melanoma or non-small cell lung cancer (NSCLC) [5]. However, the pathogenesis of VKHD-like uveitis is still unclear. Some investigators believe that ICIS-induced VKHD-like uveitis may be related to genetic factors [6]. Furthermore, previous research suggests that the immunological mechanism of anti-melanocytes may be the source of inflammation [7].

It is worth noting that previous studies have shown that the average time of onset of ocular irAEs is about 35 days, the total time range is 28.0–111.5 days [8], and the number of days of onset of uveitis associated with ICIs is 34.50 days [9]. In this case, the patient developed VKHD-like uveitis about 20 months after receiving PD-1 monoclonal antibody treatment and had no previous history of related eye diseases and no obvious prodromal symptoms before onset. In a study of ocular immune-related adverse events associated with lung cancer immune checkpoint inhibitors, no significant differences in the timing of lung cancer-induced ocular irAEs were found between different ICIs, age, sex, and race. We considered that there were differences between previous studies and the primary lesion site of this patient, which led to a long interval of onset of the disease, which may also be related to the primary site of the tumor, tumor nature, autoimmune disease susceptibility factors, and immunogenetic background. In this case, the patient did not develop eye symptoms during the first ICIs treatment, possibly because the tumor escaped the immunesurveillance and the immunotherapy, but recent surgery or chemotherapy might have led to tumor lysis and more exposure tumor antigens, and there might have been a simultaneous viral infection with T-cell activation that was aggravated by the immunotherapy. Although it is not clear why there is a difference in the timing of VKHD-like uveitis, it is important to note that VKHD-like uveitis as an eyes-related adverse event occurs not only early in the use of immune checkpoint inhibitors but also after a long period of use. For such patients, we suggest that regular ophthalmic follow-up and evaluation should be necessary along with ICIs therapy.

After the diagnosis of VKHD-like uveitis, this case was classified as a grade 4 adverse event according to the American Society of Clinical Oncology Clinical Practice Guidelines (ASCO Guidelines) for the Management of Immune-related Adverse Events in Patients, which recommended permanent discontinuation of immune checkpoint inhibitor use and urgent referral to ophthalmology and systemic corticosteroids and intravitreous, periocular, or topical corticosteroid treatment [10]. After 5 days of treatment, the patient’s visual acuity improved significantly with peripheral exudative retinal flattening. One month later, the patient’s BCVA had improved to 20/25, the retinas in both eyes were flat, and no signs of active uveal inflammation were observed. A long-term follow-up was conducted.

In summary, this is the first report of VKHD-like uveitis after treatment with a novel PD-1 antibody, Toripalimab. Our case suggests that novel PD-1 antibodies can also induce VKHD-like uveitis, and ocular irAEs after ICIs can lead to deterioration of quality of life and impact patient compliance, according to the pharmacovigilance database of the U.S. Food and Drug Administration (FDA) Adverse Event Reporting System. Therefore, the case of VKHD-like uveitis that may occur after the ICIs use cycle of more than 20 months should also be given continuous attention and timely treatment. This case provides a reference basis for identifying, diagnosing, and treating patients who have received PD-1 antibody inhibitor treatment for a long time, have no obvious ocular prodromal symptoms, and have a rapid onset of VKHD-like uveitis. It also provides a reference basis for future research on the pathogenesis of VKHD-like uveitis caused by ICIs treatment.

## Data Availability

The datasets used and analyzed during the current study are available from the corresponding author upon reasonable request.

## Abbreviations

ANA: Antinuclear antibody
ASCO Guidelines: American Society of Clinical Oncology Clinical Practice Guidelines
BCVA: Best correct visual acuity
CRP: C-reactive protein
CT: Corneal thickness
CTLA-4: Cytotoxic T lymphocyte antigen-4
ds-DNA: Anti-double-stranded DNA antibody
ESR: Erythrocyte sedimentation rate
FDA: Food and Drug Administration
FFA: Fluorescein Fundus Angiography
HBsAg: Hepatitis B surface antigen
ICGA: Indocyanine Green Angiography
ICIs: Immune checkpoint inhibitors
irAEs: Immune-related adverse events
NSCLC: Non-small cell lung cancer
OCT: Optical coherence tomography
T-SPOT: Tuberculosis infection cell spot test
VKHD: Vogt-Koyanagi-Harada disease
